# A bibliometric analysis of telerehabilitation services for patients with stroke

**DOI:** 10.3389/fneur.2022.1026867

**Published:** 2023-01-09

**Authors:** Dan Wu, Haojie Zhang, Yan Leng, Kai Li, Shijue Li, Wai Leung Ambrose Lo

**Affiliations:** ^1^Department of Rehabilitation Medicine, The First Affiliated Hospital, Sun Yat-sen University, Guangzhou, China; ^2^Guangdong Engineering and Technology Research Center for Rehabilitation Medicine and Translation, Sun Yat-sen University, Guangzhou, China

**Keywords:** stroke, telerehabilitation, rehabilitation, bibliometric analysis, cerebral vascular accident (CVA)

## Abstract

**Background:**

Routine rehabilitation services were disrupted by the COVID-19 pandemic outbreak. Telehealth was identified as an alternative means to provide access to these services. This bibliometric study aimed to analyze the scientific literature to discover trends and topics in the potential applications of telerehabilitation for patients with stroke.

**Methods:**

The Web of Science electronic database was searched to retrieve relevant publications on telerehabilitation. Bibliometric data, including visual knowledge maps of authors, countries, institutions, and references, were analyzed in CiteSpace. Visualization maps were generated in VOSviewer to illustrate recurrent keywords and countries actively involved in this research area.

**Results:**

The analysis was performed based on 6,787 publications. The number of publications peaked between 2019 and 2021, coinciding with the years of the COVID-19 outbreak. A total of 113 countries in Europe, North America, Asia, and Oceania had at least one publication in this research field, implying global attention in this research area. Nine of the top 10 most productive countries are developed countries, indicating a potentially higher capability to implement a telerehabilitation program.

**Conclusion:**

The potential benefits and diversity of telerehabilitation are already highly visible from clinical studies, and further improvements in these technologies are expected to enhance functionality and accessibility for patients. More relevant research is encouraged to understand the barriers to increased adaptation of telerehabilitation services, which will finally translate into a significant therapeutic or preventive impact.

## Introduction

Cerebral vascular disease is among the top three causes of years lived with disability worldwide. Stroke is the most common form of cerebral vascular disease that often results in deficits in motor function, cognitive function, and speech and swallowing functions (1). The rehabilitation process is often lengthy and resource intensive. During the COVID-19 pandemic, restrictions on social interactions and the closure of public facilities were in place to minimize further outbreaks (2). Routine rehabilitation services were disrupted, and healthcare providers promptly sought alternative provision models for rehabilitation services (3). Telehealth was identified as a way to increase access to these services due to its advantages of overcoming transport-related barriers, increasing the frequency of therapy sessions, individualizing interventions, and enhancing monitoring through technology (4). The term “telehealth” refers to an array of healthcare provisions that are provided to patients at a remote location (5) and was once stated by the World Health Organization (WHO) as having the potential to transform health service delivery across the world (6).

Telerehabilitation was defined as rehabilitation services delivered *via* information and communication technologies (5). It can include the provision of rehabilitation consultations *via* a phone call or a videoconference (7), the use of a computerized program (8) or a virtual reality system (9, 10) to provide home-based activities, or the provision of therapeutic devices or wearable sensors that can remotely monitor or provide feedback to the clinicians for a follow-up (11). The effectiveness in delivering rehabilitation to patients with stroke *via* telehealth remains inconclusive. Two Cochrane reviews reported that the evidence was insufficient to reach conclusions about the effectiveness of telerehabilitation but indicated it as a reasonable model of service delivery for stroke rehabilitation (12, 13). The underpinning mechanisms for telehealth rehabilitation should not differ from those observed in a clinical setting, given that it is a change of rehabilitation setting rather than a trial of different rehabilitation techniques. The benefits of telerehabilitation could be plentiful. One of the intuitive benefits is its potential to increase access to rehabilitation services. With the general increase in human lifespan and a global aging society, the WHO called for an increase in the provision of rehabilitation as a strategy to meet the Universal Health Coverage target set by the United Nations (14). In lower-middle income or developing countries, access to stroke services is often limited due to a shortage of rehabilitation staff (15). Patients in low- and middle-income countries are most likely to return home after acute care, increasing their need for home- and community-based rehabilitation services (16). Technological developments such as wearable sensors, portable training devices, or mobile health may supplement the provision of telerehabilitation after stroke. Global research and development trends of in the application of telerehabilitation after stroke remain uncertain. These uncertainties include the types of intervention, the outcome measures adopted, and the level of service adoption, as well as their reach, which were identified as barriers to the deployment of telerehabilitation for stroke services (4).

Bibliometric analysis is an analytical approach that provides an integrative view of profiling a specific scientific area. The main purpose of this bibliometric analysis is to identify core research or authors and their relationships by analyzing all publications related to a given field (17). It is an approach that explores the publication patterns, including the development trends over time and across the globe, the influence of the articles, the authors, and the journals. Bibliometric analysis relies on a large volume of data to provide abundant information on a topic, providing an understanding of the intellectual landscape of a specific topic through citation statistics, that is, the number of times they are cited in publications written by other scholars (18). It differs from a systematic review or meta-analysis as they focus on a specific research question, such as whether a specific type of intervention is effective for a specific population (19). A systematic review requires a careful analysis of the quality, quantity, and consistency of research findings (20). Findings from a systematic review provide evidence for policymakers to judge the risks, benefits, and harms of healthcare behaviors and interventions and provide a starting point for clinical practice guideline developers (21). Bibliometric analysis generates a combination of quantitative and narrative measurements, with greater emphasis on narratives to inform scholars about the trend of research and research impact in a wider context (22). It is an alternative option to a systematic review to map and visualize global research trends on telerehabilitation as part of stroke services. Instead of attempting to answer a specific question or evaluate the supporting evidence on one particular aspect, as in the case of a systematic review or meta-analysis, the central part of bibliometric analysis is the production of a bibliographic map to visualize the intellectual origins and the structure of the literature related to a specific topic over a period of time (23). Thus, bibliometric analysis was chosen to analyze the progress of research on telerehabilitation for stroke and to provide objective data on which to base future research. To date, no bibliometric analysis investigating the application of telerehabilitation after stroke has been found. The current study aimed to conduct a bibliometric analysis of the scientific literature published in the last few decades to discover trends and explore topics in the application of telerehabilitation after stroke.

## Methods

### Data source and search strategy

**Figure 1:**
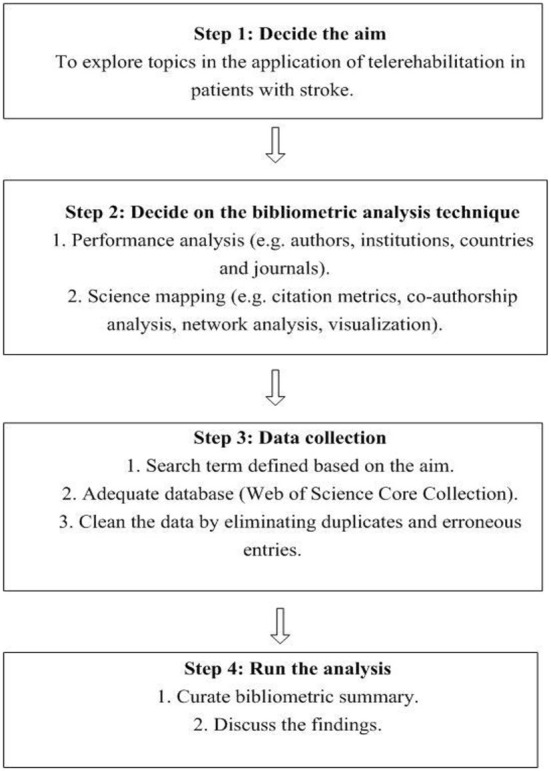
A flowchart of the study procedure.

The procedure for conducting a bibliometric analysis was in accordance with the published guidelines (24). An overview of the bibliometric analysis procedure is illustrated in Figure 1. Data were retrieved from the Web of Science Core Collection database. The Web of Science Core Collection database contains 20,000 high-quality influential journals (25). The database contains comprehensive citation index records and is appropriate for data mining and citation analysis (26). Thus, it has been widely adopted in published bibliometric studies (27–29). In addition, some of the citation metrics may vary between different databases. Citation counts were different for PubMed, Scopus, and the Web of Science, potentially due to the adequacies and different frequencies of citation updates (30) and the different number of journals indexed by each database (31). The labeling of article types also varies between databases (32). Thus, it is not feasible to combine citation metrics from multiple databases for analysis.

A literature search was conducted in June 2022 with the following search strings: ALL (“digital medicine” OR “telemedicine” OR “mHealth” OR “eHealth” OR “mobile health” OR “digital health” OR “telerehabilitation” OR “exergame” and ALL (“stroke” OR “ischemic stroke” OR “cerebral vascular accident”), and ALL (“rehabilitation” OR “hand function” OR “lower limb function” OR “cognitive function” OR “motor function” OR “gait” OR “speech” OR “fine motor skill” OR “gross motor skill”). Articles published between January 1999 and December 2021 were searched. Duplicates and erroneous titles were manually screened and removed by two researchers. The articles that did not contain the search terms were deemed to have erroneous titles and were removed from the analysis. Bibliometric data, including visual knowledge maps of authors, countries, institutions, and references, were analyzed in CiteSpace 5.3R4. The software CiteSpace is based on the Java platform and is applied to generate bibliometric data. VOSviewer was applied to produce a key term map showing phrases from the titles and abstracts of the publications. The publication records from the Web of Science Core Collection were input into VOSviewer to generate key term maps to illustrate the co-occurrence network of key terms and the collaboration network of countries. The computer software generates the diagram based on the number of occurrences of a particular key term and its co-occurrences. The size of the nodes corresponds to the number of co-occurrences of the citations. Larger nodes denote a greater number of occurrences or citations. Only key terms that appeared more than 20 times were included.

## Results

### Landscape overview

**Figure 2:**
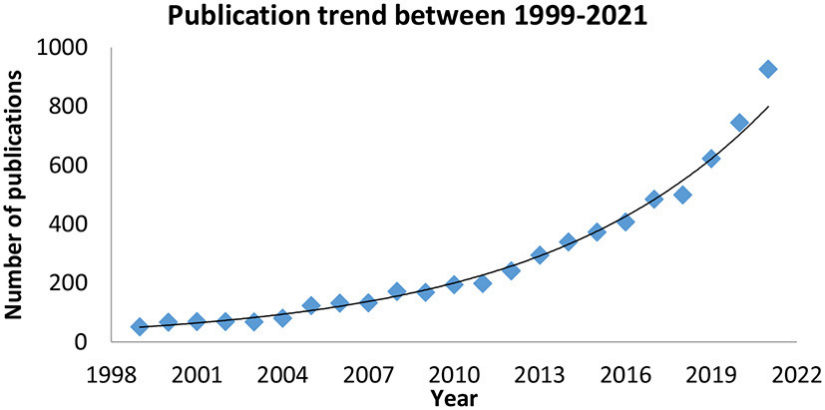
The number of publications between 1999 and 2021.

The literature search returned a total of 15,922 records. After removing duplicates and erroneous titles, 6,787 items were included. A gradual increase in the number of publications was observed throughout the years. The number of publications peaked between 2019 and 2021, coinciding with the years of the COVID-19 outbreak. Figure 2 presents a graphical illustration of the number of publications between January 1999 and December 2021.

### Most productive entities

**Figure 3:**
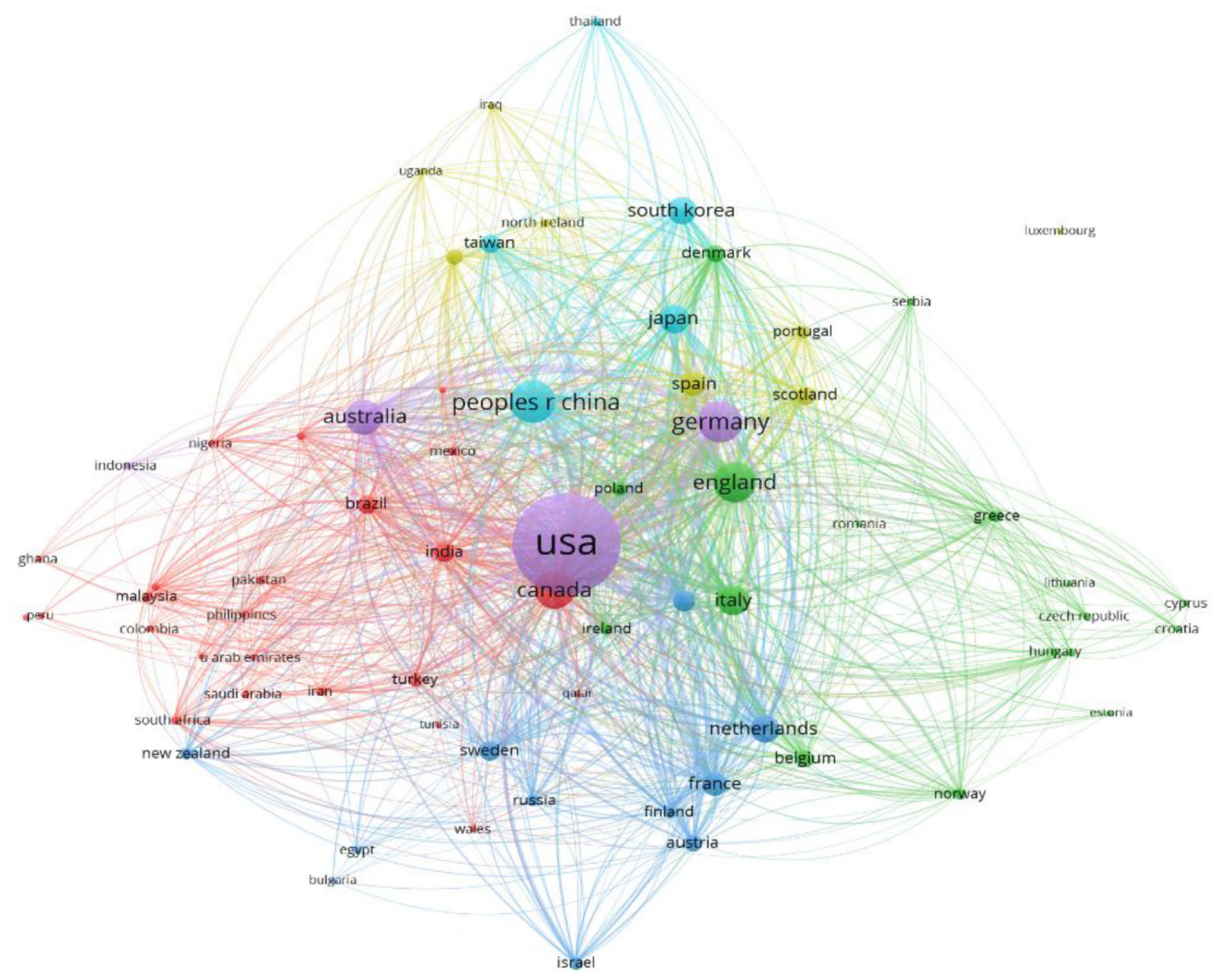
The strength of the collaboration link between each productive/involved country with more than 20 publications.

**Figure 4:**
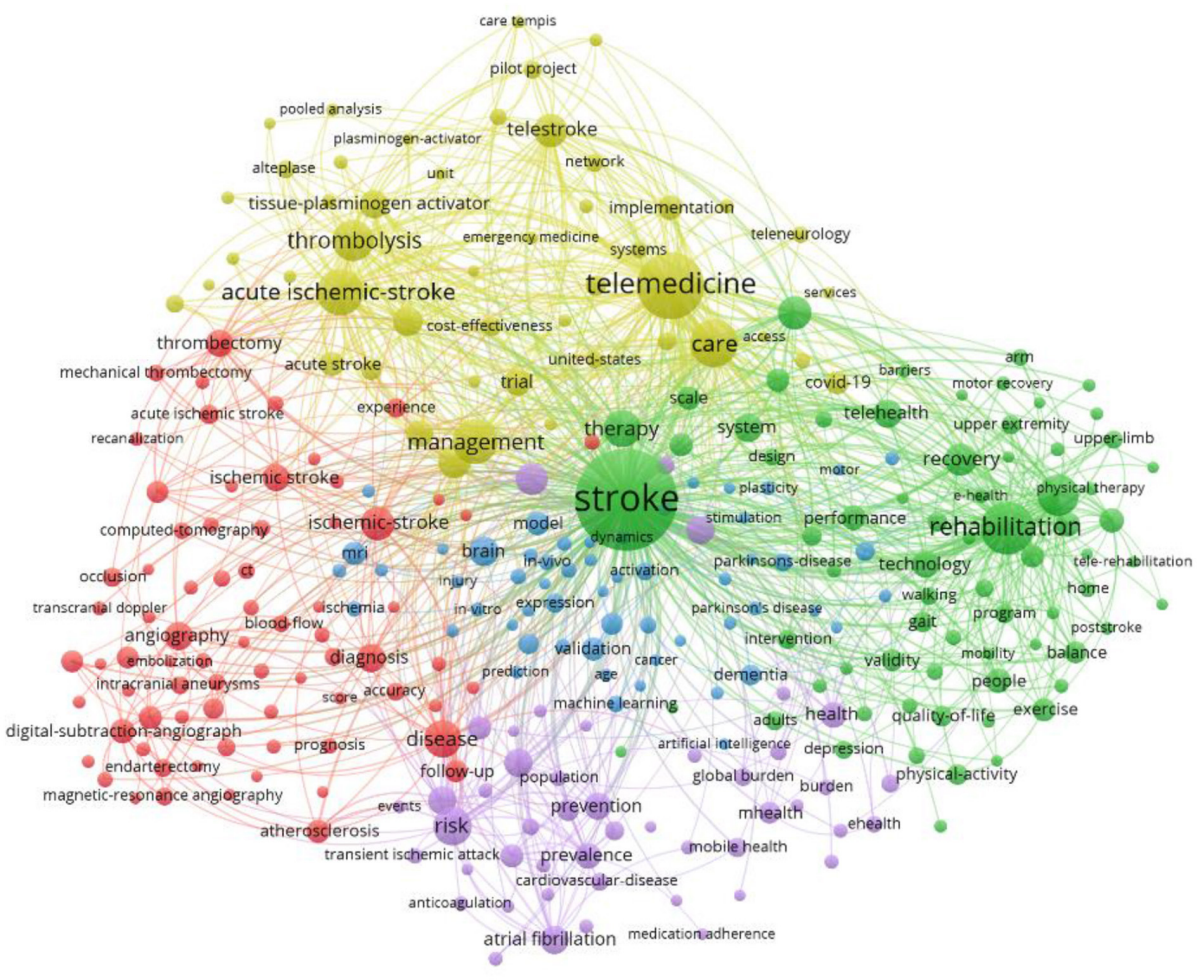
The link strength map of keywords.

A total of 113 countries had at least one publication in this research field. Figure 1 depicts the link strength of collaboration among all countries. Table 1 presents the top 10 countries that are most productive. Nine out of the 10 most productive countries are developed countries. Figure 3 presents collaboration link strength between each productive/involved country. The top 10 most productive institutes are presented in Table 2. All 10 productive countries are found in developed countries. The top 10 keywords in the published literature are presented in Table 3. The top two keywords are stroke and telemedicine, followed by telerehabilitation. The results of a keyword search might indicate that rehabilitation had not been studied as extensively as the medical aspect of stroke. The link strength of the keywords is shown in Figure 4. The 10 most productive journals are listed in Table 4. The top three journals are specialized in stroke, whereas three journals are specialized in healthcare services.

**Table 1:** The top 10 countries that are productive in this research area.

**Countries**	**Number of publications**	**Number of citations**
USA	3,264	112,707
China	617	10,076
Germany	582	15,082
England	521	16,415
Canada	463	16,217
Australia	417	8,232
Italy	290	8,234
Japan	268	5,020
South Korea	256	3,303
Netherland	253	8,051

**Table 2:** The top 10 keywords in the published literature of this specialized field.

**Keywords**	**Occurrences**
Stroke	2,188
Telemedicine	961
Rehabilitation	602
Care	489
Acute ischemic stroke	463
Management	425
Telerehabilitation	376
Thrombolysis	368
Risk	328
Disease	304

**Table 3:** The top 10 productive institutes.

**Institutes**	**No. of publication**	**No. of citations**
University of Melbourne	130	2,800
Mayo Clinic	120	2,885
University of Alabama	117	5,034
Johns Hopkins University	114	5,985
University of Californian Los Angeles	112	4,682
Stanford university	109	8,891
Massachusetts General Hospital	108	4,475
Monash university	107	1,426
University of California San Francisco	105	5,372
Harvard University	104	7,107

**Table 4:** The top 10 journals that are productive in this specialized field.

**Journals**	**No. of documents**	**No. of citations**	**Research areas**
Stroke	447	14,367	Clinical Neurology
International Journal of Stroke	163	1,377	Clinical Neurology
Journal of Stroke & Cerebrovascular Diseases	156	1,283	Neurosciences
Frontiers in Neurology	104	455	Clinical Neurology
Journal of Telemedicine and Telecare	99	1,956	Health Care Sciences and Services
Neurology	88	2,922	Clinical neurology
Telemedicine and e-Health	88	1,195	Health Care Sciences and Services
PLoS One	88	2,287	Multidisciplinary Sciences
JMIR mHealth uHealth	81	1,503	Health Care Sciences and Services
Journal of Neurologic Physical Therapy	60	710	Clinical Neurology

## Discussions

### Major findings

This bibliometric analysis analyzed 6,783 publications on telerehabilitation for patients with stroke. Telerehabilitation for stroke is gaining global attention, as evidenced by contributions to the scientific literature from Europe, North America, Asia, and Oceania. The increased use of the Internet *via* smartphones and wearable devices has been the primary driver of the application of telerehabilitation programs in the last few decades. Starting in 2019, there was a sharp increase in the number of publications that peaked in 2021, confirming the catalytic effect of the COVID-19 pandemic on the adoption of the telerehabilitation application (33).

Various applications of the telerehabilitation method have been observed throughout the years. The types of telerehabilitation interventions have been broadly divided into three categories: phone- or video-based, computer- or game-based, and mobile application-based. Phone- or video-based telerehabilitation may include the adoption of telephone services and video conference technologies to provide functional exercise and adaptive strategies for patients with stroke or their caregivers (34–40). Some studies reported a dedicated connection system to improve usability and system stability. Phone-based telerehabilitation services remain a popular method in the delivery of rehabilitation programs, likely due to their accessibility and lack of specific equipment. A computer- or game-based telerehabilitation program involves a purpose-built rehabilitation device with bespoke software or a commercial gaming device to provide a rehabilitation program that is not necessarily under supervision. Balance exercises and upper- and lower-limb functional training are common interventions delivered through computer systems (8–11, 35, 38, 41–43).

Computer-based programs were also designed for cognitive function (44, 45) and speech function (46). Mobile application-based interventions include using customized developed mobile phone programs to induce behavior change (47) and utilizing smartphones' built-in sensors to monitor upper limb motion (48).

Nine of the top 10 productive/active countries and the top 10 institutes that are productive and heavily or actively involved in this research field are in developed countries. These findings reflect the capability to implement telerehabilitation in developed countries. In contrast, it might be challenging to introduce a digital program in developing countries or underdeveloped regions with challenging environments (49). The early implementation of telerehabilitation was market driven, and some of the most successful digital healthcare programs were sponsored or driven by global telecommunications industries (50). As a result, more resources were available for research publications generated in developed countries than in developing countries. China is the world's largest developing country (51), and stroke is the leading cause of disability-adjusted life years nationally (52). The commitment to developing remote stroke services in China was witnessed through the establishment of the National Telestroke Center in 2014 to provide a nationwide network platform for stroke services. The 13th Five-Year Plan for National Economic and Social Development of the People's Republic of China called for comprehensive prevention and control of chronic diseases, which resulted in a large number of published studies on the management of stroke (53). Rapid economic growth over the past decade and its heavy investment in the development of healthcare technology to combat the rising cost of stroke care may contribute to the observed number of publications.

The incorporation of digital programs into mainstream healthcare services remains a complex problem and is a particularly challenge in developing countries (54). It requires a change in the work processes of healthcare organizations (55) and in the behaviors of patients. The data from the present study indicated that the research trend focused primarily on the clinical feasibility and effectiveness of telerehabilitation for patients with stroke. The weight of evidence tends to support the idea that the clinical benefit is at least as effective as offline consultations. The results for the searched keywords indicate that data on the perspectives of users of digital products or the potential barriers to adapting telerehabilitation for patients with stroke are minimal. Previous literature highlighted barriers to adapting telerehabilitation services, such as negative patient attitudes toward a change from traditional face-to-face consultation (56) and a lack of guidelines for requesting telerehabilitation programs (57). Other potential barriers may include the physical characteristics of the device (e.g., small text fronts and screen brightness), the terminology associated with a digital device (e.g., scrollbar, cursor, and browser) (58), the cost of adopting a digital service (e.g., Internet access and purchase of the digital device), or a slow Internet connection (59). Further research in these particular areas is warranted to facilitate the integration of telerehabilitation as part of stroke services.

## Conclusion

The findings of this study enable the identification of a major contributor in the application of telerehabilitation to stroke services and form the basis for future research. In the field of telerehabilitation for stroke services, more than 6,000 publications were found. The analyzed literature had global contributions, with the heaviest contribution coming from the USA, followed by China and Germany. The global impact of telerehabilitation supports its potential benefits and diversity, and further improvements in these technologies are expected to enhance functionality and accessibility for patients. More relevant research is encouraged to understand the barriers to the increased adaptation of telerehabilitation services, which will result in a significant therapeutic or preventive impact. Future collaboration between high- and low-income countries to identify the requirements of users from different backgrounds may facilitate the clinical translation of telerehabilitation technologies.

## Data availability statement

The raw data supporting the conclusions of this article will be made available by the authors, without undue reservation.

## Author contributions

DW and HZ were involved in the literature search and drafting of the manuscript. YL, KL, and SL were involved in the analysis, interpretation, and drafting of the manuscript. WL was responsible for the design of the study protocol, analysis, manuscript revision, and management of the study. All authors have read and approved the final manuscript and meet the four primary ICMJE authorship criteria.
